# Data-Driven Phenotyping of Central Disorders of Hypersomnolence With Unsupervised Clustering

**DOI:** 10.1212/WNL.0000000000200519

**Published:** 2022-06-07

**Authors:** Jari K. Gool, Zhongxing Zhang, Martijn S.S.L. Oei, Stephanie Mathias, Yves Dauvilliers, Geert Mayer, Giuseppe Plazzi, Rafael del Rio-Villegas, Joan Santamaria Cano, Karel Šonka, Markku Partinen, Sebastiaan Overeem, Rosa Peraita-Adrados, Raphael Heinzer, Antonio Martins da Silva, Birgit Högl, Aleksandra Wierzbicka, Anna Heidbreder, Eva Feketeova, Mauro Manconi, Jitka Bušková, Francesca Canellas, Claudio L. Bassetti, Lucie Barateau, Fabio Pizza, Markus H. Schmidt, Rolf Fronczek, Ramin Khatami, Gert Jan Lammers

**Affiliations:** From the Sleep Wake Center SEIN Heemstede (J.K.G., R.F., G.J.L.), Stichting Epilepsie Instellingen Nederland, Heemstede; Department of Neurology and Clinical Neurophysiology (J.K.G., R.F., G.J.L.), Leiden University Medical Center; Department of Anatomy and Neurosciences (J.K.G., S.M.), Amsterdam UMC (Location VUmc), the Netherlands; Center for Sleep Medicine, Sleep Research and Epileptology (Z.Z., R.K.), Klinik Barmelweid AG, Barmelweid, Switzerland; Leiden Observatory (M.S.S.L.O.), Leiden University, the Netherlands; Sleep-Wake Disorders Unit (Y.D., L.B.), Department of Neurology, Gui-de-Chauliac Hospital, CHU Montpellier; National Reference Centre for Orphan Diseases, Narcolepsy, Idiopathic Hypersomnia, and Kleine-Levin Syndrome (Y.D., L.B.); Institute for Neurosciences of Montpellier INM (Y.D., L.B.), Univ Montpellier, INSERM, France; Neurology Department (G.M.), Hephata Klinik, Schwalmstadt, Germany; Department of Biomedical, Metabolic and Neural Sciences (G.P.), University of Modena and Reggio Emilia; IRCCS Istituto delle Scienze Neurologiche di Bologna (G.P, F.P.), Bologna, Italy; Neurophysiology and Sleep Disorders Unit (R.d.R.-V.), Hospital Vithas Nuestra Señora de América, Madrid; Neurology Service (J.S.C.), Institut de Neurociències Hospital Clínic, University of Barcelona, Spain; Neurology Department and Centre of Clinical Neurosciences (K.S.), First Faculty of Medicine, Charles University and General University Hospital, Prague, Czech Republic; Helsinki Sleep Clinic (M.P.), Vitalmed Research Center, Finland; Sleep Medicine Center Kempenhaeghe (S.O.), Heeze; Eindhoven University of Technology (S.O.), the Netherlands; Sleep and Epilepsy Unit–Clinical Neurophysiology Service (R.P.-A.), University General Hospital Gregorio Marañón, Research Institute Gregorio Marañón; University Complutense of Madrid (R.P.-A.), Spain; Center for Investigation and Research in Sleep (R.H.), Lausanne University Hospital, Switzerland; Serviço de Neurofisiologia (A.M.d.S.), Hospital Santo António/Centro Hospitalar Universitário do Porto and UMIB-Instituto Ciências Biomédicas Abel Salazar, Universidade do Porto, Portugal; Neurology Department (B.H., A.H.), Sleep Disorders Clinic, Innsbruck Medical University, Austria; Department of Clinical Neurophysiology (A.W.), Institute of Psychiatry and Neurology, Warsaw, Poland; Department of Sleep Medicine and Neuromuscular Disorders (A.H.), University of Münster, Germany; Neurology Department (E.F.), Medical Faculty of P.J. Safarik University, University Hospital of L. Pasteur Kosice, Kosice, Slovak Republic; Neurology Department (M.M.), EOC, Ospedale Regionale di Lugano, Ticino, Switzerland; Department of Sleep Medicine (J.B.), National Institute of Mental Health, Klecany, Czech Republic; Fundacio d`Investigacio Sanitaria de les illes balears (F.C.), Hospital Universitari Son Espases, Palma de Mallorca, Spain; Department of Neurology (C.L.B., M.H.S., R.K.), Inselspital, Bern University Hospital, University of Bern, Switzerland; and Department of Biomedical and Neuromotor Sciences (F.P.), University of Bologna, Italy.

## Abstract

**Background and Objectives:**

Recent studies fueled doubts as to whether all currently defined central disorders of hypersomnolence are stable entities, especially narcolepsy type 2 and idiopathic hypersomnia. New reliable biomarkers are needed, and the question arises of whether current diagnostic criteria of hypersomnolence disorders should be reassessed. The main aim of this data-driven observational study was to see whether data-driven algorithms would segregate narcolepsy type 1 and identify more reliable subgrouping of individuals without cataplexy with new clinical biomarkers.

**Methods:**

We used agglomerative hierarchical clustering, an unsupervised machine learning algorithm, to identify distinct hypersomnolence clusters in the large-scale European Narcolepsy Network database. We included 97 variables, covering all aspects of central hypersomnolence disorders such as symptoms, demographics, objective and subjective sleep measures, and laboratory biomarkers. We specifically focused on subgrouping of patients without cataplexy. The number of clusters was chosen to be the minimal number for which patients without cataplexy were put in distinct groups.

**Results:**

We included 1,078 unmedicated adolescents and adults. Seven clusters were identified, of which 4 clusters included predominantly individuals with cataplexy. The 2 most distinct clusters consisted of 158 and 157 patients, were dominated by those without cataplexy, and among other variables, significantly differed in presence of sleep drunkenness, subjective difficulty awakening, and weekend-week sleep length difference. Patients formally diagnosed as having narcolepsy type 2 and idiopathic hypersomnia were evenly mixed in these 2 clusters.

**Discussion:**

Using a data-driven approach in the largest study on central disorders of hypersomnolence to date, our study identified distinct patient subgroups within the central disorders of hypersomnolence population. Our results contest inclusion of sleep-onset REM periods in diagnostic criteria for people without cataplexy and provide promising new variables for reliable diagnostic categories that better resemble different patient phenotypes. Cluster-guided classification will result in a more solid hypersomnolence classification system that is less vulnerable to instability of single features.

The classification of central disorders of hypersomnolence has been a topic of debate for decades and has been revised multiple times. This is due mainly to insufficient knowledge about the pathophysiology, reflected in a lack of validated and reliable biomarkers within this group of disorders, apart from narcolepsy with cataplexy. Different opinion articles have recently been published, all stressing the need for revision of the current classification because its application causes problems for physicians and patients when applied in daily practice.^1-6^ The current version of the International Classification of Sleep Disorders (ICSD-3) is based largely on a consensus of expert opinion and describes 3 different categories of chronic central disorders of hypersomnolence: narcolepsy type 1 (almost completely overlapping the former category called narcolepsy with cataplexy), narcolepsy type 2 (almost completely overlapping the former category called narcolepsy without cataplexy), and idiopathic hypersomnia. The disorders share the symptom of excessive daytime sleepiness, and in the absence of cataplexy and hypocretin-1 deficiency, the multiple sleep latency testing (MSLT) results and possible increased sleep duration differentiates between them. Only narcolepsy type 1 is a clinically distinct phenotype because of the specific presence of cataplexy and its strong correlation with hypocretin-1 deficiency (<110 pg/mL in the CSF).^7^

Despite these apparently clear and distinct criteria, it often proves difficult to differentiate reliably between narcolepsy type 2 and idiopathic hypersomnia. Recent studies have shown that test-retest reliability of the MSLT is poor in the absence of cataplexy, and diagnostic crossover of up to 53% was seen for narcolepsy type 2 and 75% for idiopathic hypersomnia.^8-10^ Narcolepsy type 2 may also evolve over time in some individuals; for example, individuals in whom daytime sleepiness is the sole initial manifestation may develop cataplexy many years later and thereby transition into narcolepsy type 1.^11,12^ More reliable biomarkers are needed to better differentiate between individuals with central hypersomnolence disorders, specifically in those without cataplexy. As a data-driven approach, agglomerative hierarchical clustering has previously proved useful in other diseases, objectively identifying subgroups and corresponding divisive variables by grouping people with similar characteristics in clusters.^13-17^

In this study, we used an unsupervised machine learning approach, agglomerative hierarchical clustering, to identify clusters of clinically distinct central hypersomnolence disorders. We used the advantageously large-scaled European Narcolepsy Network (EU-NN) database.^18-20^ The EU-NN is an association of 21 leading European sleep centers that launched the first prospective European web-based database for narcolepsy and idiopathic hypersomnia. One main goal of the EU-NN database is to identify new biomarkers specific to central hypersomnolence disorders and to improve definitions and understanding of its subtypes. The comprehensiveness of variables and large number of individuals across different European countries provide the opportunity to implement unsupervised machine learning algorithms in an unprecedented fashion and allows comprehensive data-driven insights into the different phenotypes of central hypersomnolence disorders.

We hypothesized that clustering would result in clear separation of individuals with the current diagnosis of narcolepsy type 1 from those without cataplexy, while mixing people currently diagnosed as narcolepsy type 2 and idiopathic hypersomnia over multiple clusters according to differences in sleep duration and presence of sleep drunkenness. Given the known poor test-retest reliability of narcolepsy type 2 and idiopathic hypersomnia diagnoses,^8-10^ our main focus was on grouping those without cataplexy.

## Methods

The analysis steps are divided into core and advanced analyses (Table 1). The core analyses are essential for understanding the clinical implications of the clustering results, whereas the advanced analyses in eAppendix 1 (links.lww.com/WNL/B970) validate why we deem the clustering results trustworthy.

**Table 1:**
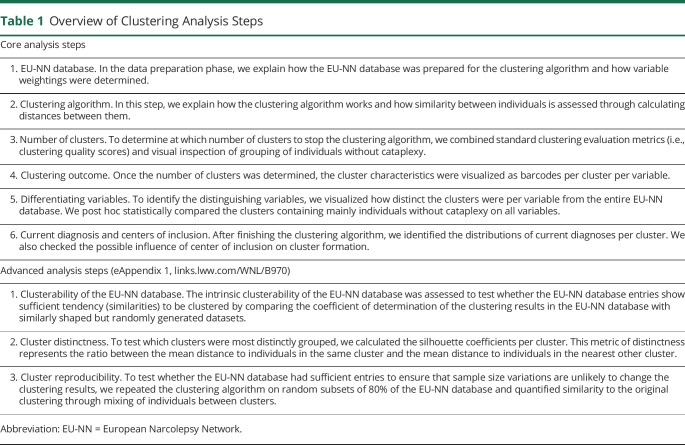
Overview of Clustering Analysis Steps

### EU-NN Database

Records of 1,078 adults and adolescents (≥13 years old) with central hypersomnolence disorders from 21 European sleep centers were included. In line with diagnostic ICSD-3 recommendations, only data of individuals unmedicated at the time of evaluation (including polysomnography and MSLT) were used. In total, 97 variables were input into the hierarchical clustering (eAppendix 2, links.lww.com/WNL/B970). Variables were assessed by sleep experts (e.g., symptom presence), objectively assessed (e.g., sleep tests, hypocretin and HLA-DQB1*0602 positivity), or self-reported through questionnaires (Epworth Sleepiness Scale and Fatigue Severity Score). Except for the questionnaire results that were fully patient rated, other subjective variables were entered by the clinician after the clinical interviews. For the database preprocessing steps, we refer to eAppendix 3.

### Clustering Algorithm

In clustering, similarity is measured by calculating a distance between individuals; similar values on the input variables result in a smaller distance. Each individual is initially a cluster of their own, and the closest individuals (or clusters) are then sequentially combined into larger clusters until there is only 1 cluster left. Details on the distance calculations are reported in eAppendix 3 (links.lww.com/WNL/B970).

### Number of Clusters

The number of clusters was determined combining 2 techniques. We first calculated multiple standard clustering evaluation metrics (i.e., clustering quality scores) that describe how well the clustering algorithm performs with different numbers of clusters (eAppendix 3, links.lww.com/WNL/B970). These metrics are normally high when individuals are similar to others in the cluster and distinct from the individuals in other clusters. The main aim of this study was to see whether data-driven algorithms would segregate narcolepsy type 1 and identify more reliable subgrouping of individuals without cataplexy because current diagnostic criteria struggle most with this subpopulation. We therefore also focused on subgrouping of individuals without cataplexy by visual inspection of the clustering steps of the full dataset from 15 to 2 clusters to better understand how people without cataplexy were subdivided and when these clusters were merged. The final model is usually a compromise of the evaluation metrics and the clinical aim of the study.

### Clustering Outcome

Clustering results were visualized as barcodes representing the mean normalized values per cluster on all variables (also called means barcodes). Variables were ordered according to the aforementioned categories, and clustering mean values were left blank when <10 values were present within a cluster.

### Differentiating Variables

Two methods were used to quantify differentiating variables between clusters. First, we used a resampling technique to test how different the clustering means were from the entire EU-NN database. We then also formally compared clusters dominated by individuals without cataplexy on all variables.

A resampling technique was implemented to test whether the clustering results deviated from the entire EU-NN database. The resampling technique enabled us to deduce the extent to which the means barcodes were different from what would be expected if the same number of clusters with the same sizes were randomly drawn from the entire EU-NN database. We generated 10,000 such draws and calculated the mean and SD of each draw per cluster per variable. For each variable, we divided the difference between the resampled mean and corresponding original clustering mean by the SD of the resampled means. This was done per variable per cluster and visualized as the significances barcodes. Only values with >25 observations were displayed. The significances barcodes better represent how substantially different the means barcodes are from the entire EU-NN database.

We post hoc statistically compared the resulting clusters with a substantial proportion of individuals without cataplexy on all included variables using Mann-Whitney tests. If the variables contained count data, we used χ^2^ tests instead. Corrected values of *p* < 0.05 were reported after multiple-comparisons correction with the Benjamini-Hochberg procedure to decrease the false discovery rate to 0.05.

### Current Diagnosis and Centers of Inclusion

Researchers were blinded to the center of inclusion and current diagnosis of the individuals until the hierarchical clustering was completed. After the clustering was finished, pie charts were generated per cluster representing the current diagnosis (with physician's diagnostic certainty) and centers of inclusion. Contingency table statistics (sensitivity, specificity, positive predictive value, and negative predictive value) were separately computed for clusters dominated by narcolepsy type 1, and narcolepsy type 2 and idiopathic hypersomnia. Centers of inclusion were shown to check whether these could have influenced the clustering. To better understand the general characteristics of the study population, the characteristics of the current ICSD-3 diagnoses are included in eAppendix 2 (links.lww.com/WNL/B970).

### Data Availability

For this study, we used the newly developed clustering package Bowerbird*,* which integrates widely used agglomerative hierarchical clustering algorithms with clustering validation methods and intuitive data visualization options. This flexible, open-source clustering package is Python based and available online.^21^ The data that support the findings of this study are available from the authors on reasonable request.

## Results

### EU-NN Database

The database included 1,078 individuals, of whom 108 were adolescents (between 13 and 18 years of age) and 970 were adults. There were 489 female and 589 male participants. Cataplexy was present in 724 and absent in 354. In line with ICSD-3 criteria, 752 people were diagnosed as having narcolepsy type 1 (646 definite, 51 probable, 33 possible, and 33 unknown diagnostic certainty), 200 as having narcolepsy type 2 (132 definite, 49 probable, 10 possible, and 9 unknown certainty), and 126 as having idiopathic hypersomnia (83 definite, 32 probable, 6 possible, and 5 unknown certainty). eAppendix 4 (links.lww.com/WNL/B970) provides an overview of the number of inclusions per center and ethical approval. The clustering algorithm and the researchers were blinded to the current diagnosis and inclusion center until the analyses were finished.

### Clustering Algorithm and Number of Clusters

The clustering evaluation metrics did not clearly favor any single number of clusters below 15 (eAppendix 5, links.lww.com/WNL/B970). This suggests that individuals in the EU-NN database are not organized according to a single number of archetypes. This means that subdivision can still result in distinct clusters but in the presence of individuals closely bordering different clusters. The number of clusters was therefore based on subgrouping of people without cataplexy because data-driven subdivision of these individuals was our main aim. A simple model with a small total number of clusters was preferred. Visual inspection of the clustering steps from 13 to 7 clusters revealed that changes occurred only in clusters with people with cataplexy and that people without cataplexy were consistently divided into the same 2 clusters. Thus, 7 was chosen as the final number of clusters.

The dendrogram showing the clustering steps from 7 to 2 clusters is included in eAppendix 5 (links.lww.com/WNL/B970). People without cataplexy were generally grouped as 1 large cluster when 6 and 5 clusters were selected. This large cluster of people without cataplexy was subsequently combined with people with cataplexy at 4 clusters. This resulted in a steep worsening of clustering evaluation metrics, indicating poorer performance below 5 clusters.

### Clustering Outcome

**Figure 1:**
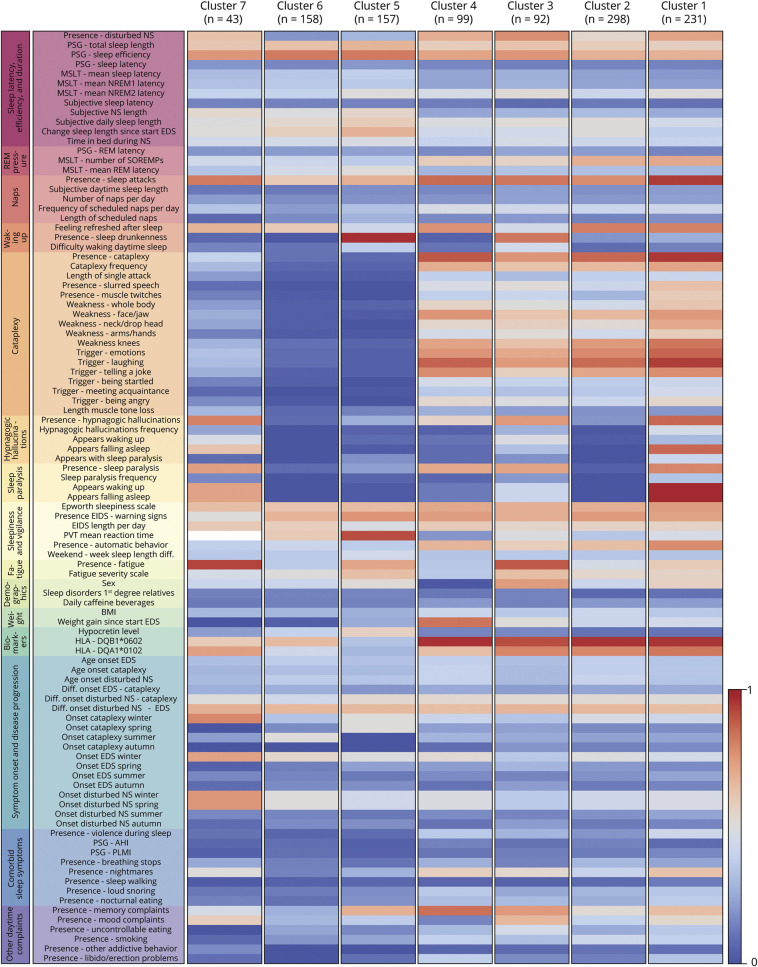
Cluster Means Barcodes Blue represents a low mean value or infrequent presence on a variable; red means a high mean value or frequent presence. Cluster sizes are displayed under the cluster number. eAppendix 2 (links.lww.com/WNL/B970) provides details on individual variables. AHI = apnea-hypopnea index; BMI = body mass index; EDS = excessive daytime sleepiness; EIDS = episodes of irresistible daytime sleep; HLA = human leukocyte antigen; MSLT = multiple sleep latency testing; NS = nocturnal sleep; PLMI = periodic leg movement index; PSG = polysomnography; PVT = psychomotor vigilance task; REM = rapid eye movement; SOREMP = sleep-onset REM period.

The means barcodes (Figure 1) show that people with cataplexy were grouped into 4 clusters (1–4) with 231, 298, 92, and 99 individuals, respectively. Those without cataplexy were grouped into clusters 5 (157 people) and 6 (158 people), and there was 1 smaller cluster (7) mixing 43 people with and without cataplexy. The variable categories nighttime sleep, difficulties in waking up, cataplexy, hypnagogic hallucinations, sleep paralysis, sleepiness, and biomarkers were most often different between clusters. eAppendix 6 (links.lww.com/WNL/B970) gives a full overview of raw variable values per cluster.

### Differentiating Variables

**Figure 2:**
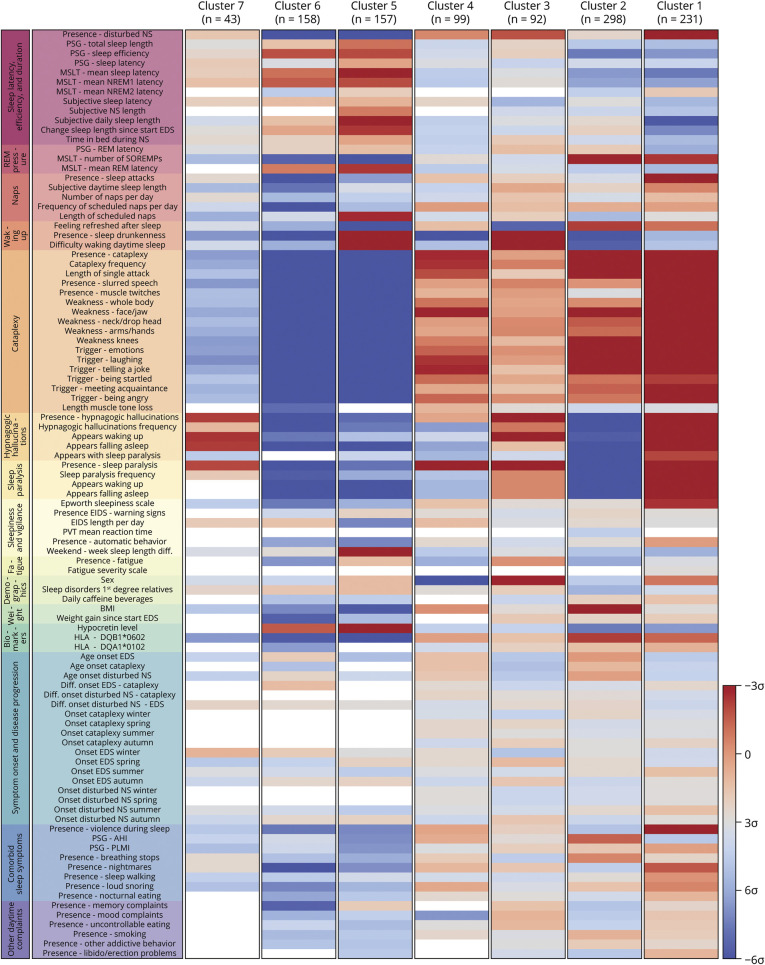
Cluster Significances Barcodes Blue represents a significantly lower value on a variable for a cluster compared to the entire European Narcolepsy Network (EU-NN) database; red means a significantly higher value. Difference with the entire EU-NN database is displayed in SDs. Cluster sizes are displayed under the cluster number. eAppendix 2 (links.lww.com/WNL/B970) provides details on individual variables. Blank fields included <25 observations. AHI = apnea-hypopnea index; BMI = body mass index; EIDS = episodes of irresistible daytime sleep; HLA = human leukocyte antigen; MSLT = multiple sleep latency testing; PLMI = periodic leg movement index; PSG = polysomnography.

From the significances barcodes (Figure 2), it is noticeable that the first 4 clusters were different mainly in noncataplectic symptoms and represented different narcolepsy with cataplexy subtypes. Cluster 1 included the most severe phenotype with frequent presence of disturbed nocturnal sleep (72%), sleep attacks (94%), cataplexy attacks (97%) that were most often complete with many potential triggers, hypnagogic hallucinations (90%), and sleep paralysis (82%). Cluster 2 was the largest but least affected cluster in which hypnagogic hallucinations (22%) and sleep paralysis (5%) were notably absent. In contrast to the other clusters of people with cataplexy, those in cluster 3 reported more difficulty in waking up (77%) with frequent presence of sleep drunkenness (83%), fatigue complaints (88%), and not feeling refreshed after sleep (36% not refreshed, 36% not always refreshed). This cluster was composed predominantly of male participants (74%). Cluster 4 mainly consisted of female participants (99%) with present (but infrequent) hypnagogic hallucinations (61%) and sleep paralysis (73%). Clusters 1 through 4 all had frequent MSLT sleep-onset REM periods (SOREMPs), HLA-DQB1*0602 positivity, and low mean hypocretin-1 levels.

People without cataplexy were generally grouped into clusters 5 and 6 (157 and 158 people, respectively). As displayed in Table 2, individuals in cluster 5 presented with significantly more sleep drunkenness, subjective difficulty in awakening, memory complaints, fatigue, and larger weekend to week sleep length difference compared to those in cluster 6. In addition, cluster 6 had a relatively higher percentage of HLA-DQB1*0602 positivity and lower hypocretin-1 concentrations. No significant differences were found between clusters 5 and 6 on MSLT-related variables and the Epworth Sleepiness Scale.

**Table 2:**
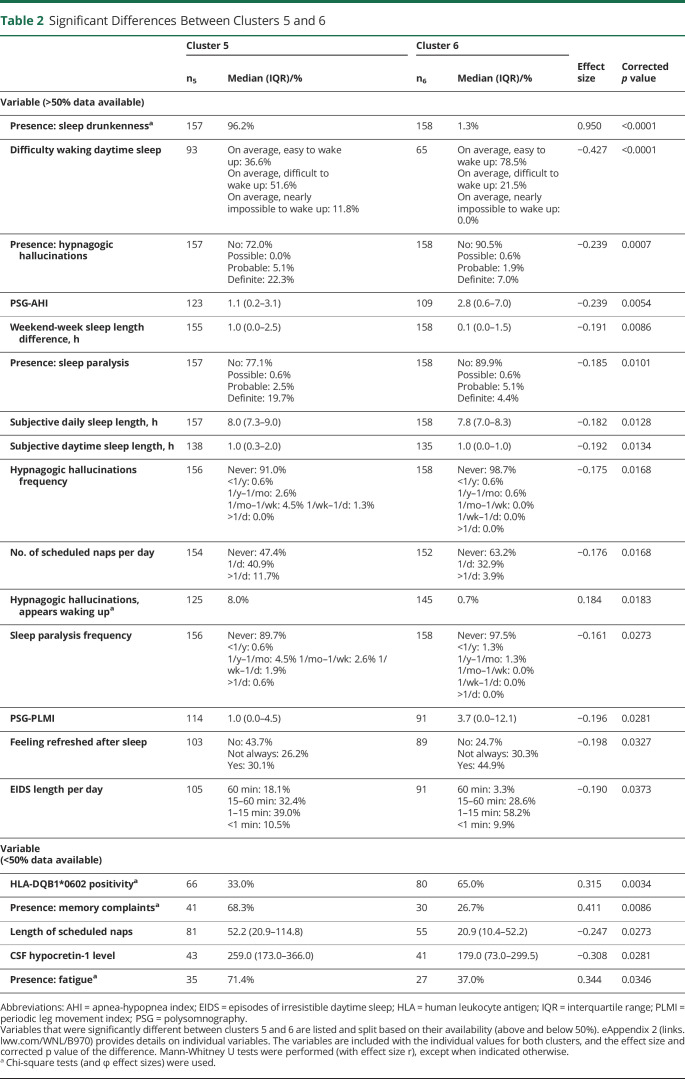
Significant Differences Between Clusters 5 and 6

Cluster 7 is a smaller cluster evenly mixing individuals with and without cataplexy. Apart from the presence of hypnagogic hallucinations and sleep paralysis, there are no clear distinguishing variables from other clusters.

### Current Diagnosis and Centers of Inclusion

**Figure 3:**
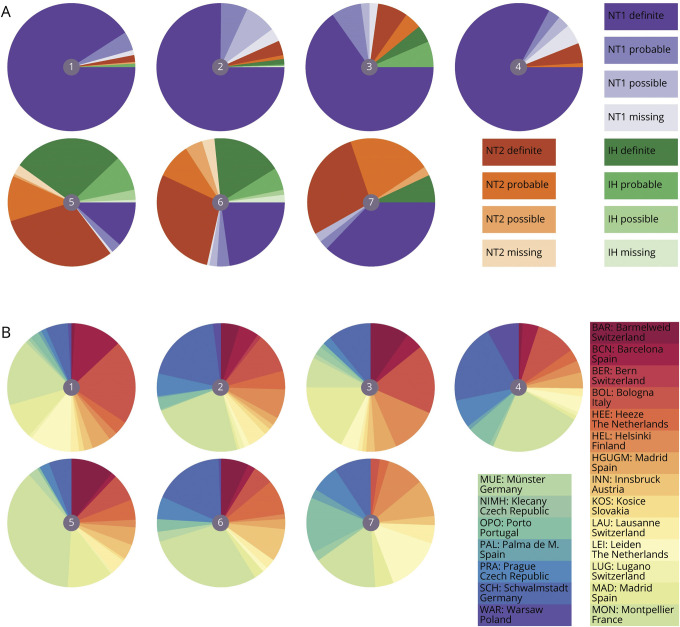
Current Diagnoses and Centers of Inclusion (A) Current diagnosis with physician's diagnostic certainty, visualized as pie charts per cluster. Central number in the pie charts corresponds to cluster identification. Clusters 1 through 4 are dominated by narcolepsy type 1 (NT1), whereas narcolepsy type 2 (NT2) and idiopathic hypersomnia (IH) are more common in clusters 5 through 7. (B) To check whether center of inclusion (or country) could have introduced bias in the clustering, distribution of the centers of inclusion is visualized as pie charts per cluster. This shows that there is no clear dominance of single centers in individual clusters and that individuals from one center are spread over multiple clusters.

Physicians were, in general, most confident in diagnosing narcolepsy type 1 and more doubtful with narcolepsy type 2 and idiopathic hypersomnia (Figure 3A). Clusters 1 through 4 included mainly people diagnosed with narcolepsy type 1 with a definite diagnostic certainty (91%, 75%, 65%, and 83%). Contingency table statistics for narcolepsy type 1 within clusters 1 through 4 showed a sensitivity of the clusters of 0.76, specificity of 0.83, positive predictive value of 0.93, and negative predictive value of 0.76. Within cluster 3, there were 18 individuals (20%) without cataplexy but with sleep drunkenness, hypnagogic hallucinations, and often sleep paralysis. Clusters 5 and 6 were dominated by people without cataplexy, and narcolepsy type 2 and idiopathic hypersomnia diagnoses were similarly mixed over the 2 clusters (cluster 5, 45% narcolepsy type 2 and 40% idiopathic hypersomnia; cluster 6, 45% narcolepsy type 2 and 27% idiopathic hypersomnia). Contingency table statistics for narcolepsy type 2 and idiopathic hypersomnia within clusters 5 and 6 showed a sensitivity of the clusters of 0.76, specificity of 0.91, positive predictive value of 0.78, and negative predictive value of 0.90. There was also a considerable proportion of individuals with narcolepsy type 1 in clusters 5 and 6 (cluster 5, 15%; cluster 6, 28%). Most of these individuals had atypical or mild cataplexy (cluster 5, 35%; cluster 6, 38%) or absent cataplexy but low hypocretin-1 levels (cluster 5, 26%; cluster 6, 33%). Cluster 7 was a mixed cluster of mainly narcolepsy types 1 and 2. Centers of inclusion were evenly spread over the clusters (Figure 3B). In eAppendix 2 (links.lww.com/WNL/B970), we present summary measures of the population using current ICSD-3 diagnoses.

## Discussion

This work presents the largest study to date on central hypersomnolence disorders that includes >1,000 adolescents and adults and the full scope of central hypersomnolence disorder characteristics. Our results provide important data-driven insights into new clinical biomarkers to improve future diagnostic criteria, especially for individuals without cataplexy. The clustering algorithm is able to identify distinct subgroups, principally separating people with cataplexy from those without. Further subdivision of those without cataplexy resulted in 2 clusters evenly mixing individuals with narcolepsy type 2 and idiopathic hypersomnia, which were separated on the basis of variables related to awakening (e.g., sleep drunkenness and subjective difficulty in awakening), sleep need (e.g., weekend-week sleep length difference), and objective biomarkers (HLA-DQB1*0602 positivity and hypocretin-1 level). MSLT parameters were not significantly different between the 2 clusters with individuals without cataplexy. The advanced cluster distinctness and resampling analyses revealed (eAppendix 1, links.lww.com/WNL/B970) that the 2 clusters of people without cataplexy were most distinctly grouped of all clusters (also from each other) and had good cluster reproducibility. People with cataplexy (generally diagnosed as having narcolepsy type 1) were further split into multiple subtypes that likely reflect different disease severities. These subtypes were different mainly in sex distribution and presence and severity of cataplexy, hypnagogic hallucinations, sleep paralysis, and sleep drunkenness.

Thanks to the large number of patients and hypersomnolence-related variables, our analyses have produced more reliable and detailed results than 2 other studies that have previously tried to identify subtypes in central hypersomnolence disorders using agglomerative hierarchical clustering.^22,23^ These studies respectively included only 96 participants or only people with idiopathic hyperomnia.^22,23^ included only people with idiopathic hypersomnia. Clustering in both studies was performed on just 7 and 3 variables, respectively. The small number of clustered variables in these studies limited their ability to identify differentiating variables among all hypersomnolence aspects.

Multiple narcolepsy experts have advocated revising the current classification, but guidance is lacking in defining new subgroups and corresponding diagnostic criteria.^1-6^ Our data-driven approach provides the opportunity to critically evaluate current ICSD-3 classification and shows that refinement of the hypersomnolence without cataplexy criteria is needed that would yield more consistent categorization. The advanced analyses in eAppendix 1 (links.lww.com/WNL/B970) revealed that the 2 clusters with people without cataplexy had good reproducibility and were most distinctly grouped compared to other clusters. The most prominent differential variables for subgrouping people without cataplexy include the presence of sleep drunkenness, subjective difficulty in awakening, mean weekend-week sleep length difference, and HLA-DQB1*0602 positivity. These differentiating symptoms suggest that certain subtypes/phenotypes of central disorders of hypersomnolence involve neuronal networks different from the cataplectic phenotype, probably mediated by other (still largely unknown) pathophysiology or comorbid conditions. Redefining key symptoms may help to establish new diagnostic methods such as forced awakening, unrestricted/extended sleep opportunity, and other biomarkers.

Sleep inertia is the complaint of difficulty in achieving complete wakefulness at the end of a sleep period, potentially accompanied by confusion, disorientation, and poor motor coordination or even ataxia. Sleep drunkenness is considered a severe manifestation of this phenomenon.^24^ Historically speaking, sleep drunkenness had already been introduced by Roth^25^ as an important differential marker when idiopathic hypersomnia was first conceptualized in the 1950s.^26,^^27^ To date, quality of awakening has consistently been undervalued by the ICSD criteria for central hypersomnolence disorders, despite multiple studies suggesting its importance in people without cataplexy.^24-32^ Besides the presence of subjective sleep drunkenness by patient history, the sensitivity and specificity of standard vigilance testing directly before and after MSLT naps could be explored to quantify the level of sleep drunkenness. The Sustained Attention to Response Task^33-35^ and Psychomotor Vigilance Test^36^ have previously been used to quantify vigilance deficits in people with central hypersomnolence disorders but not yet in relation to sleep drunkenness. A smaller study including different central hypersomnolence disorders has also previously reported event-related potentials during forced awakening to potentially quantify sleep inertia.^37^

Difficulties in waking up and increased sleep demand frequently, but not always, coexist in people without cataplexy.^27,28,30,31,38^ Classically, these individuals were grouped in the ICSD-2 diagnosis idiopathic hypersomnia with long sleep time.^39^ In our results, cluster 5 reported a substantially greater subjective weekend-week sleep length difference, a variable quantifying unfulfilled sleep need. People with increased sleep need are often unable to satisfy sleep requirements during the week because of professional/social obligations, whereas the weekend generally allows opportunity for more unrestricted sleep.^40^ The mean duration of nocturnal sleep during actigraphy may therefore not always reliably reflect the presence of an increased need for sleep. Moreover, in most sleep clinics, the MSLT routine prevents the objective confirmation of a long duration of nocturnal sleep because individuals are forced to wake early in the morning. We previously applied supervised machine learning to classify people with narcolepsy types 1 and 2 in the EU-NN database, and longer weekend-week sleep length difference was a more important deterministic parameter for narcolepsy type 2 than for type 1, further highlighting its potential to become a new clinical biomarker.^20^ It is important to keep in mind that chronic sleep deprivation and sometimes circadian disturbances also may show a similar difference. These disorders are, however, currently not included in our database. Extended 32-hour polysomnography recordings^6,41^ or actigraphy assessment of weekend-week sleep length differences during consecutive weekends may help find all potential causes for hypersomnolence without cataplexy.^40,42^ Our analyses highlight the potential of adding quality of awakening variables and weekend-week sleep length difference as sensitive new clinical biomarkers for future diagnostic criteria because they are potentially more reliable than overall mean sleep duration exclusively. Clinical interviews and questionnaires are normally used to assess these variables,^43^ but optimal quantification practices need to be validated through future studies.

Because the number of clusters was determined on the subgrouping of people without cataplexy, the differences between clusters 1 through 4 and 7 should not be overinterpreted. Clusters 1 through 4 should instead be treated as validation of the algorithm to separate people with and without cataplexy and to identify subtypes rather than strict subgroups within the narcolepsy type 1 population that likely reflect different severities. Among clusters 1 through 4, the dendrogram (eAppendix 5A, links.lww.com/WNL/B970) indicates that cluster 2 (mild severity with virtually absent hypnagogic hallucinations and sleep paralysis) is the closest to clusters 5 and 6 (clusters without cataplexy). The striking female preponderance within cluster 4, in combination with mild cataplexy, hypnagogic hallucinations, and sleep paralysis, suggests the existence of a female narcolepsy type 1 subtype. Possible sex-specific narcolepsy and the influence of hormone levels should be investigated in future studies because such mild cataplexy with fewer triggers may be easily overlooked by clinicians. The influence of sex on narcolepsy symptomatology remains essentially understudied, even though longer diagnostic delay in females individuals has previously been reported.^44^ Interpretation of cluster 7 remains difficult without evident differential variables, apart from the presence of hypnagogic hallucinations and sleep paralysis. Cluster 7 is the smallest cluster among all with poor distinctness and clustering reproducibility (eAppendix 1) and could therefore reflect an inhomogeneous group of borderline phenotypes with limited clinical relevance.

The strong differences between clusters in the significances barcodes (Figure 2) and the substantially better goodness of fit in the original clustering compared with the randomly generated datasets (eAppendix 1, links.lww.com/WNL/B970) emphasize the possibility of identifying distinct subgroups within the larger central hypersomnolence population. Lack of a clear clustering evaluation metrics peak suggests that, independently of the number of subgroups, there will always be people with central hypersomnolence who are difficult to categorize, especially when strict cutoffs are used (e.g., during the MSLT). This impression matches our clinical experience, and our results suggest that the introduction of diagnostic certainties in new diagnostic criteria could better depict confidence levels in diagnosing hypersomnolence subtypes. This idea has recently been proposed by different European experts^3^ and should be further investigated in future studies.

In addition to direct implications on current classification, the organization of the hypersomnolence disorders in multiple clusters offers opportunities for new hypothesis testing on disease etiology, disease progression, and treatment effects. Longitudinal studies will provide the opportunity to see whether some individuals are prone to change from 1 cluster to another due to either the natural course of the disease or the effects of medication. This could provide important insights into prestages of narcolepsy and data-driven treatment regimens for newly diagnosed individuals. More frequent HLA-DQB1*0602 positivity and lower hypocretin-1 levels were, for example, seen in cluster 6 than in cluster 5, suggesting a pathophysiologic nature of hypersomnolence complaints closer to narcolepsy type 1. Individuals within cluster 6 may be more likely to show disease progression and should therefore be closely monitored for development of cataplexy.

A major strength of the EU-NN database is the harmonized prospective data acquisition protocol, resulting in a true Pan-European collaboration with minimal inclusion site–specific biases. This is supported by the uniform distribution of inclusion sites over the clusters (Figure 3B). We included both adolescents and adults to best incorporate different stages of disease in our clustering analyses. Post hoc testing indicated a similar distribution of age at evaluation over different clusters. Children <13 years of age were, however, not included and should be studied in light of our proposed clusters in future studies. Even though the EU-NN database covers most important hypersomnolence-related aspects, incomplete availability of nonmandatory variables related to vigilance, cognitive functioning, and mood has hindered their full integration into our clustering analyses. Future studies should focus on these variables in relation to our proposed subgroups.

Our analyses cannot be considered fully unbiased because agglomerative hierarchical clustering algorithms require manual input of variable weightings. We tried to overcome this issue by carefully designing the analysis strategy with predetermined weighting and grouping of variables, potentially to give every asset of central hypersomnolence disorders a fair chance of influencing the clustering. Post hoc testing to determine the influence of clustering settings by resampling with random subsets of the entire database (eAppendix 1, links.lww.com/WNL/B970) showed that the EU-NN database is adequately sized with solid cluster (and biomarker) reproducibility for people without cataplexy. Separate post hoc analyses were performed to test whether our methodologic choices could have influenced the results. Clustering was repeated 3 times by excluding those with cataplexy and/or hypocretin deficiency (<110 pg/mL), with uniform weightings for all variables, and by recoding the polysomnography REM latency variable to polysomnography SOREMP presence. All 3 analyses demonstrated grouping and differentiating variables for subgrouping of those without cataplexy similar to those in the full dataset (sleep drunkenness, subjective difficulty in awakening, and weekend-week sleep length difference). These robustness checks further validate that the differentiating variables for those without cataplexy are reproducible.

Within the EU-NN database, no data entries are available for people with other conditions that could lead to daytime sleepiness complaints such as chronic sleep deprivation, myalgic encephalomyelitis/chronic fatigue syndrome, and circadian rhythm disorders. These disorders could function as control groups in future clustering analyses on central hypersomnolence disorders to test the specificity of our results. Narcolepsy type 1 could also be considered a control group with a distinct cataplectic phenotype. The fact that the clustering algorithm recognized narcolepsy type 1 as separate clusters while we were blinded for the current diagnosis provides an important argument that the algorithm is able to identify distinct, clinically relevant subgroups. Future studies should focus on external validation of our clustering results in a substantially sized independent dataset and prove internal validation by standardized follow-up data. Both approaches will validate the clinical impact of our clustering results by assessing how cluster assignments relate to clinical decisions such as treatment planning, prognosis, and mechanisms of disease.

We report an exceptionally sized quantitative subgroup assessment in people with central hypersomnolence disorders using the full range of clinical and diagnostic variables. Our study further illustrates the urgent need for new biomarkers in central hypersomnolence disorders that allow robust subclassification and improve our understanding of disease etiology. The main finding is not the number of clusters but the fact we found subgrouping consistent with current diagnosis of narcolepsy type 1, not type 2 or idiopathic hypersomnia. Instead, people with narcolepsy type 2 and idiopathic hypersomnia were divided over 2 distinctly separated clusters, differing mainly on clinical variables related to quality of awakening, including presence of sleep drunkenness and feeling refreshed after daytime sleep, weekend-week sleep length difference, and HLA-DQB1*0602 positivity. Subdivision of these individuals based only on absolute sleep duration or presence of SOREMPs is not supported by our findings. Introduction of new clinical biomarkers such as sleep drunkenness and weekend-week sleep length difference provides necessary opportunity to develop improved diagnostic criteria for people without cataplexy. At its very best, new data-driven classification of hypersomnolence disorders with levels of certainty would result in a reproducible, holistic classification system that better identifies borderline individuals and is less susceptible to volatility in single features.

## Glossary

EU-NN: European Narcolepsy Network
ICSD-3: International Classification of Sleep Disorders, 3rd edition
MSLT: multiple sleep latency testing
SOREMP: sleep-onset REM period
